# Supplementary material for: Long-Term Administration of Vespa velutina nigrithorax Venom Ameliorates Alzheimer’s Phenotypes in 5xFAD Transgenic Mice
Source: Toxins (Basel). 2023 Mar 6;15(3):203. doi: 10.3390/toxins15030203 (PMC10057037; doi:10.3390/toxins15030203)
Supplement: Supplementary file 1 [file toxins-15-00203-s001.zip › toxins-2224156-supplementary.pdf]

toxins

Article

Long-Term Administration of *Vespa velutina nigrithorax* Venom Ameliorates Alzheimer's Phenotypes in 5xFAD Transgenic Mice

Yoon Ah Jeong, Hyun Seok Yun, Yoonsu Kim, Chan Ho Jang, Ji Sun Lim, Hyo Jung Kim, Moon Bo Choi, Jae Woo Jung, Jisun Oh and Jong-Sang Kim

Supplementary Figures

(A)

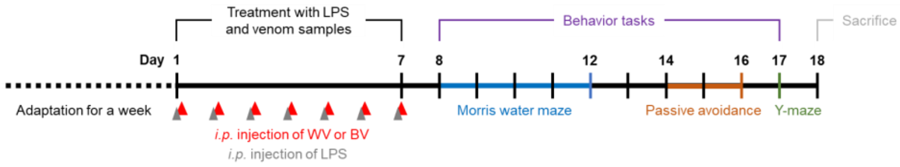

(B)

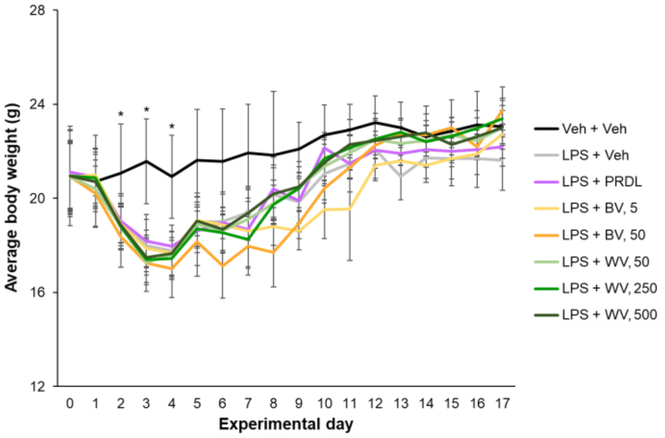

(C)

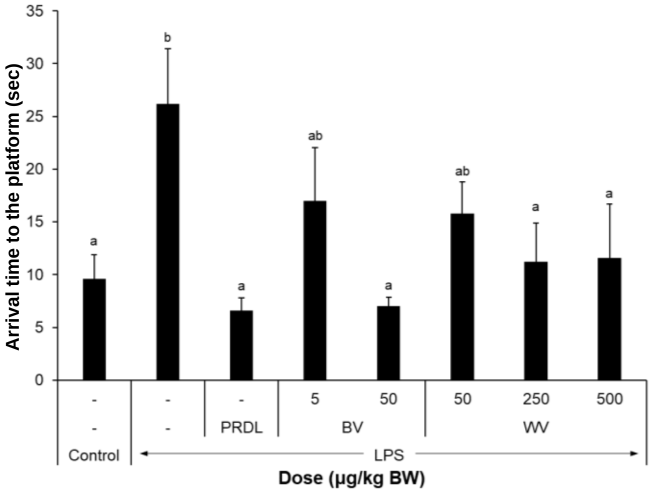

(D)

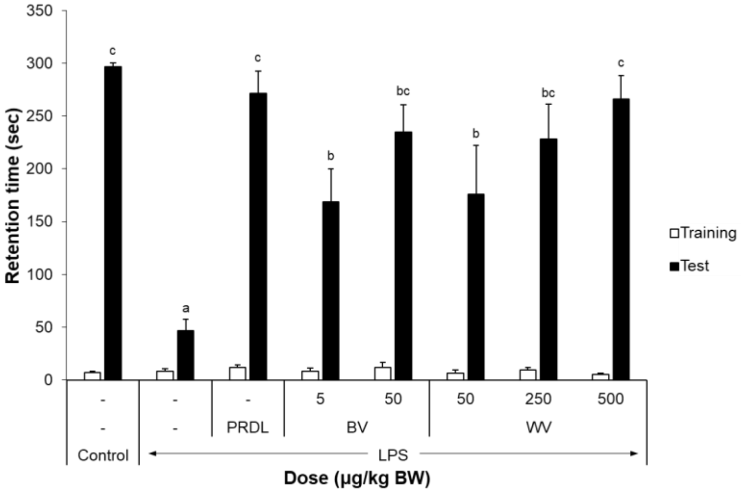

Figure S1. Wasp venom improved learning and memory in lipopolysaccharide (LPS)-treated wild-type C57BL/6J mice. A total of 48 WT mice (C57BL/6J, 6-week old, male) were assigned to 8 groups (six mice per group) and treated as follows: (1) Vehicle with no LPS, (2) 1 mg/kg BW/day LPS and vehicle, (3) LPS and 10 mg/kg BW/day prednisolone, (4) LPS and BV at 5 μg/kg BW/day, (5) LPS and BV at 50 μg/kg BW/day, (6) LPS and WV at 50 μg/kg BW/day, (7) LPS and WV at 250 μg/kg BW/day, and (8) LPS and WV at 500 μg/kg BW/day. BW, body weight; PRDL, prednisolone; BV, bee venom; WV, wasp venom. **(A)** Experimental scheme. LPS and venom (WV or BV) were administered to WT C57BL/6J mice by intraperitoneal injection only for the first 7 days, followed by behavioral tasks for the next 10 days. **(B)** Body weight changes over the experimental period. The average BW was significantly decreased for the first 4 days after LPS treatment. **(C)** Spatial learning and memory as assessed by the MWM. **(D)** Associative learning and memory as assessed by the PAT. Values are expressed as mean ± SD (n = 6). Values marked with different letters are statistically significant difference from each other at $p < 0.05$.

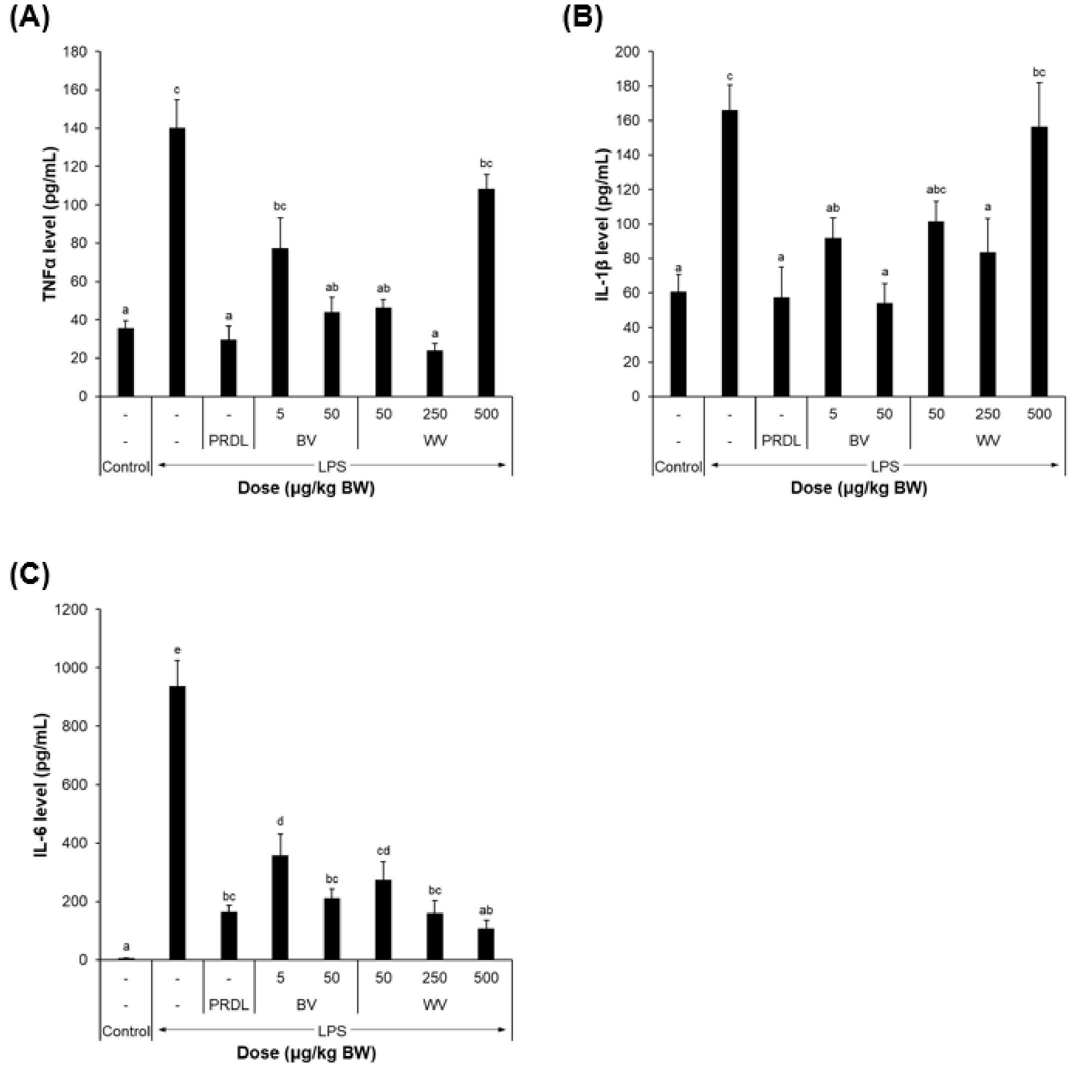

Figure S2. Wasp venom reduced pro-inflammatory cytokine expression at the mRNA level in the hippocampus of LPS-treated WT mice. **(A–C)** SYBR green-based reverse transcription quantitative PCR (RT-qPCR) was performed on total RNA isolated from hippocampal tissue. The relative mRNA expression levels of *TNFα* (A), *IL-1β* (B), and *IL-6* (C) were quantitatively determined by normalizing to the mRNA expression level of *β-actin*. RT-qPCR; reverse transcription quantitative polymerase chain reaction; *TNFα*, tumor necrosis factor alpha; *IL-1β*, interleukin 1beta; *IL-6*, interleukin 6. Values are expressed as mean ± SD (n = 6). Values marked with different letters are statistically significant difference from each other at $p < 0.05$.

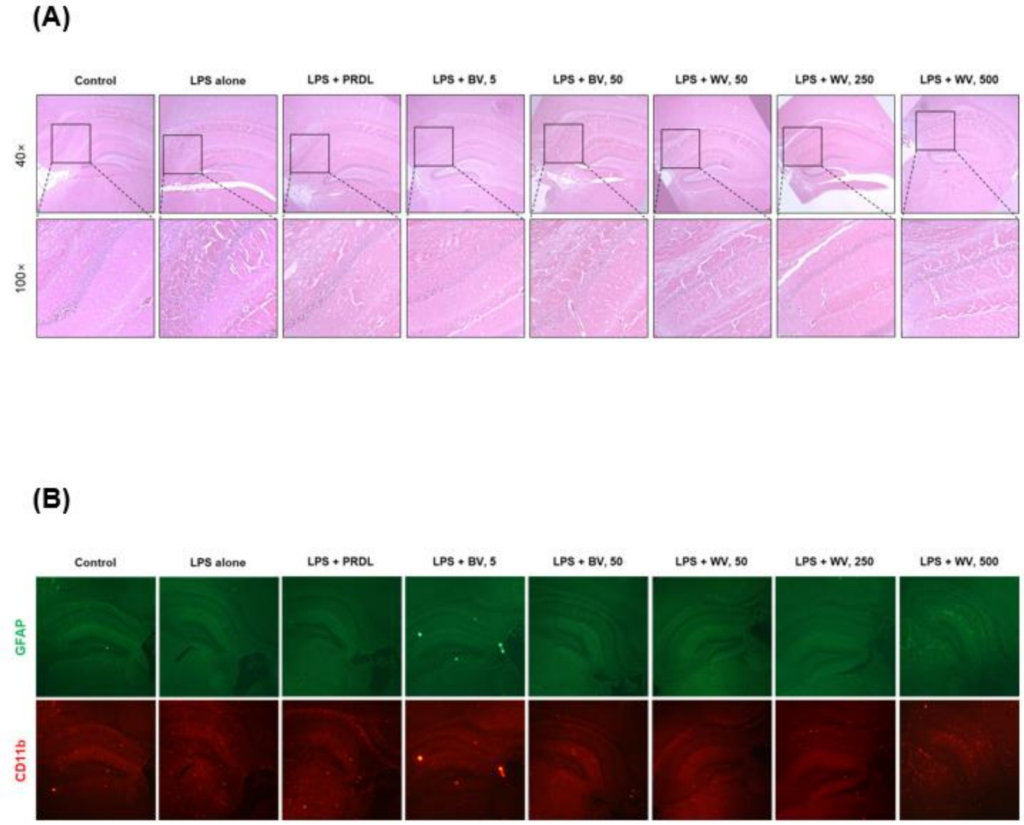

Figure S3. Wasp venom ameliorated histological damage and reduced inflammatory glial cell numbers in the hippocampal area of LPS-treated WT mice. **(A–B)** Representative images of brain slices stained with H&E (A) and antibodies against GFAP (a marker for reactive astrocytes, green) and CD11b (a marker for activated microglia, red) (B). WV, wasp venom; LPS, lipopolysaccharide; H&E, hematoxylin and eosin; GFAP, glial fibrillary acidic protein.

(A) C99 and BACE1 expressions in the hippocampus

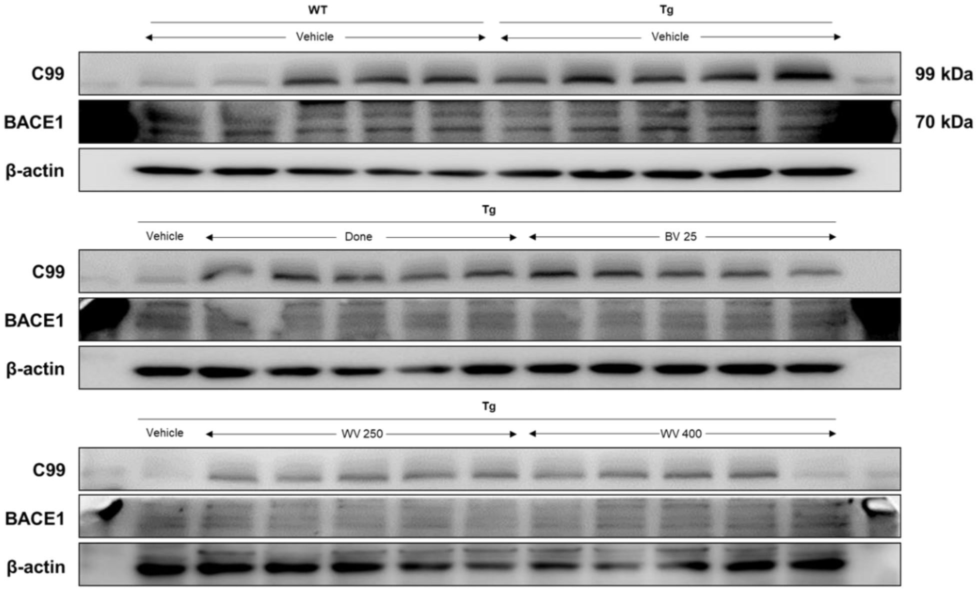

(B) COX-2 expression in the hippocampus

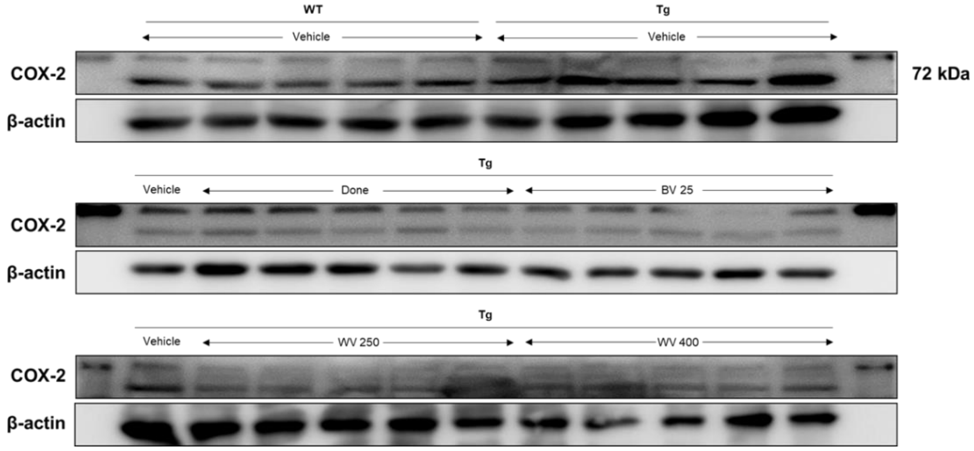

(C) COX-2 expression in the cerebral cortex

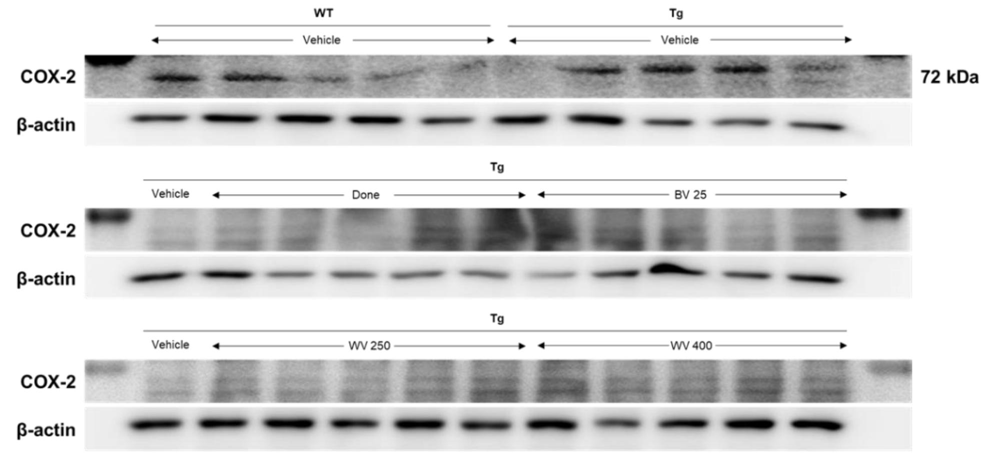

(D) NF-κB expression in the cerebral cortex

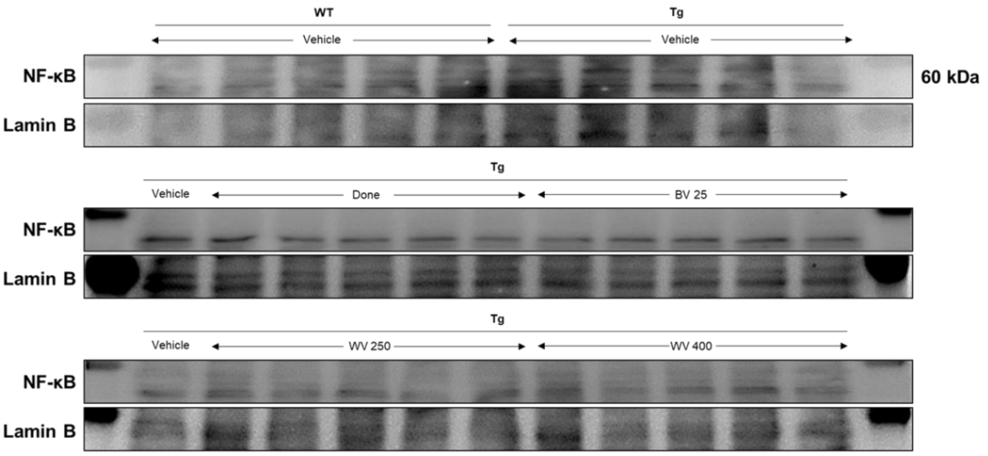

Figure S4. Western blot bands form all tissue homogenates. **(A–B)** Cytoplasmic C99, BACE1, and COX-2 protein levels in the hippocampal tissue homogenates. **(C–D)** Cytoplasmic COX-2 and nuclear NF-κB protein levels in the cerebrocortical tissue homogenates. WT, wild-type; Tg, transgenic; Done, donepezil; BV, bee venom; WV, wasp venom; C99, C99 fragment, 99 amino acids long C-terminal APP fragment; BACE1, beta-secretase; COX-2, cyclooxy-genase 2; NF- κB, nuclear factor kappa-light-chain-enhancer of activated B cells.
